# The Prince of Wales's Fund and St. Mark's Hospital

**Published:** 1900-02-24

**Authors:** 


### THE PRINCE OF WALES'S FUND AND ST. MARK'S HOSPITAL.

We publish the following correspondence between Sir Savile Crossley and Mr. Richard Martin, which has appeared in the *Times*:—

Sir,—The following has appeared in the Press:—

"At the 64th annual meeting of the subscribers of St. Mark's Hospital, held yesterday at the Mansion House under the presidency of Mr. R. B. Martin, M.P., the following resolution, moved by Mr. T. M. Merriman, seconded by Mr. E. F. Carey, and supported by the chairman, was unanimously agreed to:—

"'That this meeting of governors of St. Mark's Hospital wish to place on record their feelings of deep disappointment and concern that this hospital has again this year, not only been deprived of any participation in the Prince of Wales's Fund, but that the committee of such fund should have seen fit to publish in their report a statement that they consider the financial position of this hospital does not seem to require help this year, the fact being that the hospital is urgently in need of funds, and has only been able to meet the year's expenditure by the employment of legacies received during the year, amounting to upwards of £570, and the balance of the amount derived from the festival dinner in 1898, and, therefore, by publishing their opinion as above the aforesaid committee has done a serious injustice to the hospital and lessened its chance of obtaining in the future the increased assistance it so sorely needs.'"

May we state that no grant was given to St. Mark's by the Prince's Fund because the committee considered that their financial position did not then seem to require further help?

The printed accounts of the hospital show the ordinary income at £4,986, the ordinary expenditure £3,429, extraordinary income (legacies) £804. Extraordinary expenditure *nil*.

This leaves a total surplus of £2,362. In addition to this a further sum received from the festival dinner is not included in the above income.

By desire of the Council of this Fund we therefore wrote to the hospital on December 29th, 1899, suggesting that the remaining closed beds might be reopened. It is to be hoped that this suggestion may now be considered by the authorities of the hospital.—Your obedient servant,

Savile Crossley, Honorary Secretary Prince of Wales's Hospital Fund for London.

February 15th.

Sir,—Sir Savile Crossley is not a professional accountant, and he should therefore have taken extra trouble to properly investigate the financial position of St. Mark's Hospital before he issued the letter that he has circulated to the Press, which is not only contrary to fact, but on the face of it absurd. Both he and I are on the Council of the Hospital Sunday Fund, and if he had taken the trouble to look into the careful analysis of hospital accounts made by that Fund he would have saved casting that reflection on the wisdom of the administration of the Prince of Wales's Fund under which it will now labour.

In any doubtful point the Hospital Sunday Fund always invites the hospital concerned to offer such explanation as they may wish to do, so that there never can be any argument about facts. I recommend the administrators of the Prince of Wales's Fund to do the same. In the accounts of St. Mark's Hospital for 1898 the total income is given at £5,791. Included in this are :—

1. £1,025. Given by a lady to be invested for the endowment of one bed in perpetuity.

2. £1,720. Part of the sum raised at a festival dinner got up for the express object of getting funds to replace the £25,000 sold out of our investments in order to rebuild the hospital.

Neither of these sums should, for the purposes of the Prince of Wales's Fund, have been included in ordinary income.

3. Legacies over £100 which should be invested: Mrs. Mitchell, £350; Mr. Taylor, £225: Mr. Weldon, £200—£3,520, which, if deducted from £5,791, will leave an amount of £2,271 as our total spendable income for 1898, against an expenditure of £3,429 for that year, a deficiency of £1,158.

For 1899 the accounts show spendable revenue, £1,900; small legacies, £189—£2,089, against expenditure, £4,019, or a deficiency of £1,930.

For the years 1898-99 we have overspent our income by drawing on capital to the amount of £3,088.

I bring in the 1899 account, not yet published, to show the continued hardship under which the hospital suffers.

I cannot occupy your space by longer details. I have shown that the administrators of the Prince of Wales's Fund have done a grave injustice to the hospital due to their not having taken the trouble to obtain accurate information, and I ask you to say if it is likely that the committee of a hospital would appeal to the public for funds if they had wherewith to pay for their unopened beds, or to keep up their establishment.

St. Mark's Hospital is now a model hospital, fitted with every known appliance to alleviate suffering, and will well repay a visit from charitable persons.—I am, Sir, your obedient servant,

Richard B. Martin, Treasurer, St. Mark's Hospital.

10, Hill Street, Mayfair, W., Feb. 17th.

